# Cytosolic Sensors for Pathogenic Viral and Bacterial Nucleic Acids in Fish

**DOI:** 10.3390/ijms21197289

**Published:** 2020-10-02

**Authors:** Miriam Mojzesz, Krzysztof Rakus, Magdalena Chadzinska, Kentaro Nakagami, Gouranga Biswas, Masahiro Sakai, Jun-ichi Hikima

**Affiliations:** 1 Department of Evolutionary Immunology, Institute of Zoology and Biomedical Research, Faculty of Biology, Jagiellonian University, Gronostajowa 9, 30-387 Krakow, Poland; miriam.mojzesz@doctoral.uj.edu.pl (M.M.); magdalena.chadzinska@uj.edu.pl (M.C.); 2 Department of Biochemistry and Applied Biosciences, Faculty of Agriculture, University of Miyazaki, Gakuenkibanadai-nishi 1-1, Miyazaki 889-2192, Japan; gc16024@student.miyazaki-u.ac.jp (K.N.); m.sakai@cc.miyazaki-u.ac.jp (M.S.); 3 Kakdwip Research Centre of ICAR-Central Institute of Brackishwater Aquaculture, Kakdwip, South 24 Parganas, West Bengal 743347, India; g.biswas@icar.gov.in

**Keywords:** RLR, RIG-I, DExD/H-box RNA helicases, cGAS, LSm14A, PKZ

## Abstract

Recognition of the non-self signature of invading pathogens is a crucial step for the initiation of the innate immune mechanisms of the host. The host response to viral and bacterial infection involves sets of pattern recognition receptors (PRRs), which bind evolutionarily conserved pathogen structures, known as pathogen-associated molecular patterns (PAMPs). Recent advances in the identification of different types of PRRs in teleost fish revealed a number of cytosolic sensors for recognition of viral and bacterial nucleic acids. These are DExD/H-box RNA helicases including a group of well-characterized retinoic acid inducible gene I (RIG-I)-like receptors (RLRs) and non-RLR DExD/H-box RNA helicases (e.g., DDX1, DDX3, DHX9, DDX21, DHX36 and DDX41) both involved in recognition of viral RNAs. Another group of PRRs includes cytosolic DNA sensors (CDSs), such as cGAS and LSm14A involved in recognition of viral and intracellular bacterial dsDNAs. Moreover, dsRNA-sensing protein kinase R (PKR), which has a role in antiviral immune responses in higher vertebrates, has been identified in fish. Additionally, fish possess a novel PKR-like protein kinase containing Z-DNA binding domain, known as PKZ. Here, we review the current knowledge concerning cytosolic sensors for recognition of viral and bacterial nucleic acids in teleosts.

## 1. Introduction

The innate immune response relies on the recognition of evolutionarily conserved pathogen components, termed as pathogen-associated molecular patterns (PAMPs), through the germ line-encoded pattern recognition receptors (PRRs) [1]. Till now five major groups of PRRs have been discovered: (i) Toll-like receptors (TLRs), (ii) nucleotide-binding oligomerization domain (NOD)-leucin rich repeats (LRR)-containing receptors (NLRs), (iii) retinoic acid-inducible gene 1 (RIG-1)-like receptors (RLRs or RIG-1-like helicases-RLH) which belongs to the large family of DExD/H-box RNA helicases, (iv) C-type lectin receptors (CLRs) and (v) cytosolic DNA sensors [2,3,4,5,6,7]. They can be found associated to subcellular compartments–cellular and endosomal membranes, in the cytosol, as well as extracellularly and in secreted forms present in the bloodstream and interstitial fluids [8].

Interestingly, pathogens/PAMPs of quite different biochemical composition, structure and location can be recognized by slightly different yet surprisingly similar and overlapping mechanisms by limited range of host PRRs [1]. Moreover, in specific cases, PRRs also recognize self-danger signals (DAMPs, damage/danger associated molecular patterns), which are present in abnormal locations or atypical molecular complexes as a consequence of infection/inflammation, or cellular stress [9,10].

## 2. Viral and Bacterial PAMPs and Their PRRs

PAMPs are molecular structures (glycoproteins, lipopolysaccharides (LPS), peptidoglycans (PGN), and nucleic acid motifs) characterized by being invariant among entire classes of pathogens, essential for the survival of the pathogen, and distinguishable from “self” [2]. Viruses possess several structurally diverse PAMPs, including surface glycoproteins, DNA, and different kinds of RNA including viral double-stranded RNA (dsRNA) and single-stranded RNA (ssRNA) [11]. They can be present in the infecting virion or may be produced during viral replication.

Main PRR family recognizing viral glycoproteins are membrane TLRs such as TLR2 and TLR4. Thus, TLR2 has been shown to recognize for example the hemagglutinin (HA) protein of measles virus [12], while TLR4 recognizes fusion protein of respiratory syncytial virus (RSV) and envelope proteins of murine leukemia virus [13,14]. In turn, ssRNA oligonucleotides containing guanosine- and uridine-rich sequences from RNA viruses are recognized by endosomal TLR7/8, while dsRNA is recognized by TLR3 which in human is localized in endosomal membrane or cell surface. In turn endosomal TLR9 binds bacterial and viral CpG DNA [11]. Moreover, dsRNA (short), 5′-triphosphate RNA and dsRNA (long, at least 2 kbp) are recognized by RLRs (RIG-I and MDA5, respectively), whereas DAI and AIM2, from cytosolic DNA sensor family of receptors, recognize viral DNA localized in cytoplasm [2]. Recently, also NLRs have been shown to recognize viral PAMPS e.g., NLRC2 was found to recognize ssRNA from RSV, influenza A virus, and parainfluenza virus [15].

One of the most well-known bacterial PAMPs is lipopolysaccharides (LPS) of Gram-negative bacteria recognized by Toll-like receptor 4 (TLR4), however many other Gram-negative bacteria PAMPs have been described. They include peptidoglycans, flagellin, porins, unmethylated CpG DNA derived from bacterial genomes and classical B-form double-stranded (ds) DNA present in the cytosol [4]. In turn, the major PAMPs of Gram-positive bacteria are: glycolipid lipoteichoic acid anchored in the cytoplasmic membrane as well as lipoproteins embedded in the bacterial cell wall, and similarly to those of Gram-negative bacteria are: peptidoglycans (e.g., peptidoglycan-derivative muramyl dipeptide, MDP) and CpG DNA [16]. Bacterial DNA is mainly recognized by endosomal TLR9, cytoplasmic NLRP3 as well as RIG-I, AIM2-like receptors and DNA helicases, while peptidoglycans bind to TLR2 and activate NLRs (NOD2 and NALP1/3). Moreover, TLR2 recognizes lipoteichoic acid and lipoproteins of Gram-positive bacteria [11,17], while flagellin of the flagellated Gram-negative bacteria, activates TLR5 [18] and NLRC4 (also known as IPAF) [19].

Interestingly many fish species possess a higher number of functional TLR genes as compared to human (10 TLRs) or mouse (12 TLRs). In case of some TLRs it is due to presence of duplicated copies of TLRs in fish genome but there are also several fish-specific TLRs not present in mammals. These are for example TLR22 which is present on the cell surface and sense dsRNA outside the cells or endosomal TLR21, present also in chicken, sensing CpG-oligodeoxynucleotides [20,21]. Since the essential immune mechanisms, receptors and pathways are often well conserved in vertebrates, teleost fish represent a relevant model for the study of the core immune mechanisms activated by viral infections. As presence, function and evolution of Toll-like receptors in fish has been well described previously [20,22,23,24], in the present review we mainly focus on DExD/H-box RNA helicases and intracellular DNA sensors that recognize viral and bacterial nucleic acids in cytosol.

## 3. DExD/H-Box RNA Helicases

DExD/H-box RNA helicases are conserved proteins present in all eukaryotic and prokaryotic cells but only in a minority of virus families [25]. The DExD/H-box RNA helicases consist of at least eight conserved motifs (I, Ia, Ib, II, III, IV, V, VI) which are involved in ATP binding and hydrolysis (motifs I, II and VI), nucleic acid binding (motifs Ia, Ib, IV and V) and helicase activity (motif III) [26,27,28]. Additionally, DEAD-box, but not DEAH-box helicases, have motif Q, which is necessary for the efficient binding of ssRNA as well the conformation changes that are driven by nucleotide binding and ATP hydrolysis [26].

All described conserved motifs of the DExD/H-box RNA helicases are clustered in a central core region that contains about 350 to 400 amino acids [26]. Crystal structural analysis indicated that DExD/H-box RNA helicases have two covalently linked RecA-like globular domains, each containing five β-strands surrounded by five α-helices [29]. Motifs Q, I, Ia, Ib, II and III belong to RecA-like domain 1, while motifs IV, V, and VI are present in RecA-like domain 2 [26].

DExD/H-box RNA helicases are involved in a variety of biological processes. They are implicated in all aspects of RNA metabolism, including transcription, splicing, nuclear export of mRNA, ribosome biogenesis, RNA turnover, initiation of translation, modulation of complex RNA structures and RNA degradation [30,31,32]. Many of them are multifunctional and have additional diverse roles, for example, in cell cycle regulation [33], regulation of apoptosis [34,35], cancer development/progression [36,37] and in the antiviral innate immune response. The main DExD/H-box RNA helicases involved in sensing of viral RNAs are retinoic acid-inducible gene I (RIG-I)-like receptors [38,39].

### 3.1. RIG-I-Like Receptors (RLR)

#### 3.1.1. Receptor Structure

RIG-I-like receptors include three members of cytosolic receptors: (i) retinoic acid-inducible gene I product (RIG-I) also known as DEAD box polypeptide 58 (DDX58), (ii) melanoma differentiation-associated antigen 5 (MDA5) also known as interferon induced with helicase C domain 1 (IFIH1), and (iii) laboratory of genetics and physiology 2 (LGP2) also known as DExH box polypeptide 58 (DHX58) [39].

**Figure 1:**
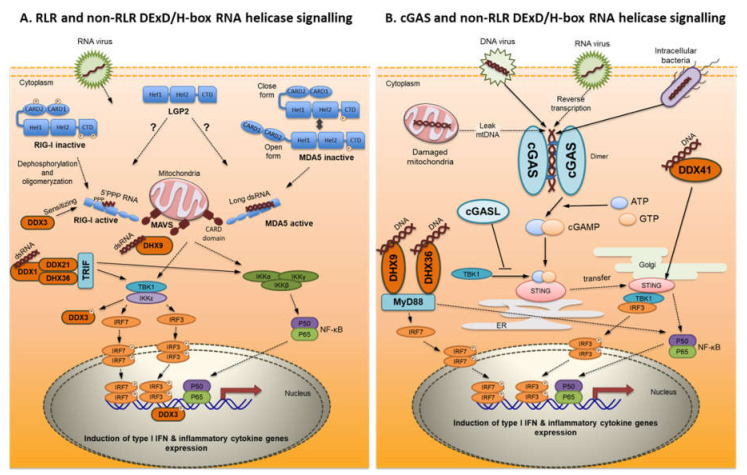
RLR (**A**), cGAS-STING (**B**) and non-RLR DExD/H-box RNA helicases (**A**,**B**) pathways detect cytoplasmic nucleic acids and activate type I IFN and pro-inflammatory cytokines. Dotted lines represents pathways confirmed in fish. (**A**) RIG-I and MDA5 upon binding of viral RNA induce signal transduction in MAVS-dependent manner leading to the induction of the expression of type I IFNs and pro-inflammatory cytokines. LGP2 does not possess signaling CARD domains and its role in the mediating of antiviral response is unclear. LGP2 receptor has been shown to function as a positive or negative regulator of RLR-mediated signaling. Viral RNA in cytoplasm is also recognized by non-RLR DExD/H-box RNA helicases: DDX3 has been proposed to sensitize RLR to sense viral RNA. It acts as a signaling intermediate downstream of TBK1 and IKKε and as a transcriptional regulator of the *ifnb* promoter. DHX9 was shown to sense dsRNA and to signal in a MAVS-dependent manner. A complex of DDX1-DDX21-DHX36 has been suggested to detect dsRNA and to signal in a TRIF-dependent manner. This leads to induction of the expression of type I IFNs and pro-inflammatory cytokines. (**B**) Upon recognition of virus or bacterial-derived DNA released into the cytoplasm, viral DNA synthesized by reverse transcription, and upon infection-induced mitochondrial dsDNA damaged and leaked cGAS synthesizes 2′3′-cGAMP. 2′3′-cGAMP binds to STING in the ER and causes its translocation to the Golgi system. STING activates TBK1-IRF3 and NF-κB and induces the production of type I IFNs and pro-inflammatory cytokines. DNA in cytoplasm is also recognized by non-RLR DExD/H-box RNA helicases: DDX41 upon binding of cytoplasmic DNA signals in a STING-dependent manner while DHX9 and DHX36 upon binding of cytoplasmic DNA signal in a MyD88-depending manner. This leads to induction of the expression of type I IFNs and pro-inflammatory cytokines.

All three proteins possess a C-terminal domain (CTD) which is responsible for binding of viral RNA, however in unbound form it functions as a repressor domain (RD) to keep the receptor in an inactive conformation. The CTD of RIG-I and MDA5 possess a positively charged groove that binds RNA and is structurally different between the two proteins. This might explain the differences in binding of RNA ligands between RIG-I and MDA5 [40]. Next, there is a flexible hinge region and the DExD/H-box RNA helicase region that consists of the RecA-like Hel1 and Hel2 domains with ATP binding and hydrolyzing activity. This receptor region is connected by another flexible hinge region to two N-terminal caspase activation and recruitment domains (CARDs), which are not present in LGP2 [37,40] (Figure 1A). CARD domains are important for their signaling by interacting with a downstream adaptor molecule-mitochondrial antiviral-signaling protein (MAVS), also known as IFNβ-promoter stimulator-1 (IPS-1), virus-induced signaling adapter (VISA), or CARD adapter inducing interferon-beta (CARDIF) [41].

In the absence of viral RNA, RIG-I and MDA5 exist in cytoplasm of the cell in a phosphorylated and “auto-inhibited” conformation where CARD domains are not accessible. In this inactivated conformation, the CARD1 domain of RIG-I is phosphorylated at residue S8, whereas the CARD2 domain is phosphorylated at residue T170 by protein kinase C-α/β (PKC-α/β) [40]. Additionally, the CTD of RIG-I is phosphorylated at residues S854, S855, and T770 by casein kinase β (CKβ) [40]. The CTD of RIG-I is also acetylated at residue K909, and its deacetylation is necessary for activation of RIG-I [42]. In contrast, the MDA5 is phosphorylated only at the CTD at residue S828 by RIO kinase 3 (RIOK3) and also by different kinases that are still unknown [40].

After ligand binding, RIG-I unfolds into an open and activated state [40]. Open conformation is made due to changes in the flexible hinge regions between CARD domains and the helicase domain as well as between helicase and CTD domain [40]. In contrast, MDA5 exists in a conformational balance between open and closed states. However, closed conformation is favored during the absence of ligand [40] (Figure 1A).

#### 3.1.2. Ligands

RLRs recognize various types of viral RNAs, and synthetic double-stranded RNA (poly(I:C); polyriboinosinic:polyribocytidylic acid) [7]. RIG-I has the highest affinity to short 5′tri-phosphorylated dsRNA [40]. It can also bind 5′di-phosphorylated dsRNA, 5′tri-phosphorylated ssRNA with a polyuridine signature and low-molecular-weight poly(I:C) (around 300 bp) [2,40,43]. The binding of 5′mono-phosphorylated dsRNA by RIG-I is uncertain since some of the studies demonstrated that 5′monophosphate dsRNA did not activate RIG-I [44] while other studies have shown that RIG-I can bind 5′monophosphate dsRNA to a certain degree [45]. MDA5 preferentially binds long dsRNA (>3 kb) and high-molecular-weight poly(I:C) (around 4–8 kbp) [40,43,46]. It has been observed that RIG-I can recognize viral nucleic acids of positive-strand RNA viruses like Japanese encephalitis virus, but also negative-stranded RNA viruses like influenza viruses, bunyaviruses, filoviruses, and rhabdoviruses [40]. On the other hand, MDA5 mainly recognizes positive-stranded RNA viruses, such as picornaviruses and arteriviruses [40]. Despite the fact that LGP2 does not contain the N-terminal CARD, it still can bind varied dsRNAs irrespective of 5′-PPP or RNA length [47].

#### 3.1.3. Signal Transduction

RLRs are broadly expressed in most tissues where they are responsive for signal transduction and activation of antiviral response in a variety of cell types [41]. Ligand binding results in receptor dephosphorylation and ubiquitination [3]. Following dephosphorylation of the RIG-I CTD, this domain is ubiquinated at residues K849 and K851 by the E3 ubiquitin ligase RIPLET. Similarly, dephosphorylation of the RIG-I CARD domain led to polyubiquitination of this domain at residue K172 by the E3 TRIM25 ubiquitin ligase [40]. It has been shown that RIG-I complexes with ubiquitin are strong inducers of type I IFNs [48]. After activation, RIG-I receptor oligomerizes with other RIG-I/dsRNA complexes to form helical oligomers and MDA5 oligomerizes to form long RNA-associated filaments [40]. When the receptors are activated and oligomerized, the CARD domain becomes exposed and interacts with CARD-containing adaptor molecule MAVS [2]. MAVS is located on the outer membrane of the mitochondria and coordinates the activation of two signaling pathways. It can activate the kinases TBK1 and IKK-ε which phosphorylate interferon-regulatory factor 3 (IRF3) and 7 (IRF7). The phosphorylated IRF3 and IRF7 dimerize and translocate to the nucleus to initiate transcription of type I IFNs which upon secretion signal in autocrine and paracrine fashion to induce expression of the interferon-stimulated genes (ISGs) among which there are many antiviral proteins, such as Mx proteins, viperin and ISG-15. MAVS can also activate the kinase complex (IKK-α, IKK-β, IKK-γ/NEMO) which leads to the phosphorylation and subsequent degradation of IκB, which in the unphosphorylated form is coupled to NF-κB. This results in NF-κB translocation to the nucleus and activation of the expression of the genes encoding pro-inflammatory cytokines [2,3,7,49] (Figure 1A).

LGP2 does not possess signaling CARD domains and its role in antiviral response induction is unclear. Strong activation of LGP2 expression occurs upon viral infection, poly(I:C) stimulation and IFNα treatment [47]. It is intriguing that in various experiments, LGP2 receptor has been shown to function as a positive or negative regulator of RLR-mediated signaling. For example, Yoneyama and co-workers [50] reported that LPG2 acts as a negative regulator of RIG-I/MDA5-mediated signaling, by interfering with the viral RNA recognition by RIG-I and MDA5. Another report suggested that LGP2 inhibits antiviral activation by engaging in a protein complex with MAVS [51,52]. In contrast, studies on LGP2-deficient mice indicated that lack of this gene resulted in deficient type I IFN response [53,54].

## 4. RLRs in Fish

The orthologs of RLRs have been identified from many fish species and fish cell lines, and their constitutive expression was demonstrated [22,43] (Table 1). Interestingly, RIG-I has been identified mainly in Cypriniformes, Siluriformes, and Salmoniformes [43], but is absent in fish of superorder Acanthopterygii, such as medaka (*Oryzias latipes*), Japanese pufferfish (*Takifugu rubripes*), tetradon (*Tetradon nigroviridis*), three-spined stickle-back (*Gasterosteus aculeatus*), European seabass (*Dicentrarchus labrax*), gilt-head seabream (*Sparus aurata*), and mandarin fish (Chinese perch; *Siniperca chuatsi*) [43]. It is unclear why RIG-I has been lost in some fish species but this gene does not occur also in some other higher vertebrate species, for example in chicken [55], and Chinese tree shrew [56].

RLRs in fish and mammals are structurally similar at the protein level [22]. However, some differences can be found at the genetic level in the exon/intron organization. For example, Japanese flounder (*Paralichthys olivaceus*) LGP2 gene contains 12 exons, while human LGP2 contains 14 exons [57]. Furthermore, LGP2 of grass carp (*Ctenopharyngodon idella*) possesses one more α-helix domain in the RD, compared with that in human LGP2 [58].

### 4.1. RIG-I (DDX58)

Intracellular localization of RIG-I demonstrated its presence in the cytoplasm of fish cells [70,73]. Up-regulation of the expression of *rig-I* after viral infection or poly(I:C) stimulation in cell lines (in vitro studies) or various organs of different fish species (in vivo studies) has been demonstrated and is summarized in Table 1. Moreover, different RIG-I isoforms were identified in several fish species. For example, *rig-I* in zebrafish (*Danio rerio*) possesses four different transcripts: *rig-Ia, rig-Ib, rig-Ic* and *rig-Id* [108]. Zebrafish *rig-Ib* encodes the typical form of RIG-I, while *rig-Ia* encodes protein with an insertion of 38 amino acids in the second CARD domain [73], *rig-Ic* encodes a protein that lacks the first 189–192 amino acid region just behind the second CARD domain, while *rig-Id* encodes a protein that lacks 2 amino acids just behind the second CARD domain, but has an insertion of 3 amino acids in the helicase domain [108]. Comparison of the constitutive expression of zebrafish *rig-Ia* and *rig-Ib* in the ZF4 cell line indicated a higher level (of about 20 folds) of *rig-Ib* expression [108]. Moreover, their expression was significantly up-regulated during spring viremia of carp virus (SVCV), and *Edwardsiella tarda* infection, although, the up-regulation of *rig-Ib* was to a lesser degree [73]. Two isofoms of RIG-I: AjRIG-Ib and AjRIG-Ibv were described in the Japanese eel (*Anguilla japonica*) [70]. Based on the structure, AjRIG-Ib represents the classical RIG-I form containing all characteristic domains, while AjRIG-Ibv is a truncated form that lacks C-terminal domain (CTD) [70]. The transcripts of both RIG-I isoforms were detected in all studied tissues/organs of the Japanese eel, with higher level of *rig-Ib* expression [70]. I.p. injection of Japanese eel with poly(I:C) induced up-regulation of the expression of both *rig-Ib and rig-Ibv* in all examined tissues/organs with more rapid up-regulation of *rig-Ibv* (as early as 8 h post-injection) than *rig-Ib* [70]. Two isoforms of the RIG-I have also been identified in gibel carp (*Carassius gibelio*) (RIG-Ia and RIG-Ib) and the genes encoding both isoforms were up-regulated during *Carassius auratus* herpesvirus (CaHV) infection in head kidney, spleen and liver [65].

Several functional studies demonstrated that knock-down of gene encoding RIG-I impairs the antiviral response in fish. Moreover, it was demonstrated that functional blockade of RIG-I by using dominant negative mutants of this receptor (RIG-I-DN) significantly attenuated in fish SVCV-induced activation of IFN promoters, and up-regulation of IFNs and ISGs in Epithelioma Papulosum Cyprini (EPC) cells, derived from fathead minnow (*Pimephales promelas*) [109]. In zebrafish ZF4 cell line, morpholino-induced knock-down of RIG-I resulted in reduction of the expression of group II type I IFNs (*ifnφ2* and *ifnφ3*), but not group I type I IFNs (*ifnφ1*), upon nervous necrosis virus (NNV) infection [71]. Several groups studied the function of RIG-I using overexpression approach. Biacchessi and co-workers [110] demonstrated that overexpression of N-terminal fragment (first 275 aa) of RIG-I in EPC cell line resulted in the induction of expression of genes encoding type I IFN and several ISGs and provided cell protection from viral hemorrhagic septicemia virus (VHSV) infection. Moreover, the authors were able to detect the presence of IFN in supernatant collected from EPC cells transfected with expression vector for RIG-I Nter (coding N-terminus protein) and this supernatant was able to protect fresh EPC cells against VHSV infection [110]. Overexpression of the two isoforms of RIG-I of the Japanese eel (*ajrig-ib* or *ajrig-ibv*) in the EPC cells led to the activation of type I IFN promoter [70]. In case of zebrafish, luciferase assay demonstrated that transfection of EPC cells with vector encoding *rig-Ib* activated the type I IFN promoter, which was not the case for *rig-Ia.* Furthermore, overexpression of *rig-Ib*, but not *rig-Ia*, reduced the viral titer during SVCV infection in EPC cells [73]. Overexpression of *rig-I* in crucian carp (*Carassius carassius*) blastulae embryonic cells (CABs) also induced activation of type I IFN promoter as demonstrated by luciferase assay [111]. In turn, Chen and co-workers [112], using expression vectors, demonstrated the role of different domains of grass carp RIG-I in downstream signaling pathway in *Ctenopharyngodon idella* kidney cell lines (CIK) cells during grass carp reovirus (GCRV) infection and stimulation with various PAMPs of viral and bacterial origin. The CARDs and helicase domains were shown to play a role in signaling cascade after GCRV infection while the CARD domains were also involved in the induction of signal transduction upon poly(I:C) stimulation. Helicase domain mediated induction of signaling pathway upon LPS and PGN stimulation and CARDs domain strengthened this induction. In all cases, RD domain inhibited activation of the signal transduction. Interestingly, lack of CARDs domain showed positive modulation in RIG-I signal transduction [112].

### 4.2. MDA5

Up-regulation of the expression of *mda5* after viral infection or poly(I:C) stimulation in cell lines (in vitro studies) or various organs of different fish species (in vivo studies) have been demonstrated and this data are summarized in Table 1. Similar to RIG-I, intracellular localization of MDA5 demonstrated its presence in the cytoplasm of fish cells [78,79,88,92].

In zebrafish, two isoforms of MDA5 (*mda5a* and its shorter splicing variant *mda5b*) were identified [92], of which *mda5a* gene consists of 16 exons and 15 introns, whereas *mda5b* does not have part of exons 11 and 13 and complete exon 12. *Mda5b* has a premature stop codon in exon 11 which results in lack of C-terminal regulatory domain (RD) embedded within the C-terminal domain (CTD) [92]. Both variants were significantly up-regulated in ZF4 cells during SVCV or *E. tarda* infection with *mda5a* showing a higher level of expression in comparison to *mda5b* [92].

A few functional studies demonstrated that inhibition of MDA5 impairs the antiviral response in fish. Zebrafish dominant-negative *mda5* mutants lacking CARD domains were more susceptible to snakehead rhabdovirus (SHRV) infection and showed reduced *ifnφ1* up-regulation during SHRV infection as compared to wild-type larvae. This effect was rescued by overexpression of *mda5* [113]. Functional blockade of *mda5* in the EPC cells, by using dominant negative mutants of MDA5 (MDA5-DN), significantly attenuated in fish activation of IFN promoters and up-regulation of IFNs and ISGs during SVCV infection [109]. Transient silencing of *mda5* in Japanese flounder gill cells (FG) by siRNA technology resulted in reduced antiviral activity upon poly(I:C) stimulation [114]. The role of MDA5 in antiviral response of fish was also studied using the overexpression approach. Overexpression of crucian carp *mda5* induced activation of type I IFN promoter in CABs cells [111] while overexpression of rainbow trout (*Oncorhynchus mykiss*) *mda5* enhanced *mx* gene expression in rainbow trout gonad 2 cells (RTG-2) [78]. Moreover, overexpression of *mda5* in hirame natural embryo (HINAE) cell line derived from Japanese flounder embryo, resulted in the inhibition of replication of hirame rhabdovirus (HIRRV), VHSV and infectious pancreatic necrosis virus (IPNV) [85]. Furthermore, HINAE cells with *mda5* overexpression showed enhanced type I IFN response during VHSV infection as compared to the cells transfected with empty vector [85]. The overexpression of zebrafish *mda5a* and *mda5b* induced strong activation of type I IFN promoter in EPC cells with *mda5a* exhibiting stronger induction [92]. The protective effect of *mda5* overexpression against viral infection was demonstrated by reduced cytopathic effect (CPE) in zebrafish liver cells (ZFL) upon SHRV infection and EPC cells upon SVCV or GCRV infection [79,113]. Other studies indicated that overexpression of orange-spotted grouper *mda5* in Grouper spleen (GS) cells delayed the CPE progression during Singapore grouper iridovirus (SGIV) or red spotted grouper nervous necrosis virus (RGNNV) infection [88]. It also induced type I IFN and IFN-stimulated response element (ISRE) promoter activities as shown by reporter gene assay and enhanced the expression of *irf3* and *irf7*. Furthermore, GS cells with *mda5* overexpression exhibited higher expression of pro-inflammatory cytokines such as *tnf-α* (during SGIV and RGNNV infection) and *il-8* (during RGNNV infection) as compared to cells transfected with empty plasmids [88]. Wan and co-workers [115] using the stable transfected CIK cell line demonstrated that grass carp MDA5 induced a stronger type I IFN response as compared to RIG-I during GCRV infection and poly(I:C) stimulation. Interestingly, the stable overexpression of *rig-I* decreased the expression level of endogenous *mda5* while the opposite effect was not demonstrated [115]. Gu and co-workers [116] studied the function of MDA5 domains using different overexpression vectors and demonstrated that helicase domain was essential in response to GCRV infection and PGN stimulation. Moreover, CARD domain alone was sufficient to induce signaling cascade after LPS stimulation [116].

### 4.3. LGP2

LGP2 was identified in many fish species and its up-regulation of the expression after viral infection or poly(I:C) stimulation in cell lines (in vitro studies) or various organs of different fish species (in vivo studies) is summarized in Table 1. The intracellular localization of LGP2 demonstrated its distribution in the cytoplasm [78,98,99] and also in nucleus [117].

Two variants of LGP2 (LGP2a and alternatively spliced LGP2b) have been identified in rainbow trout where LGP2b is 54 amino acids shorter than LGP2a due to the presence of an unspliced intron at the 3′ end region of the ORF causing early termination of translation [78]. In zebrafish, three splicing variants of the LGP2 have been described, including a full-length LGP2 (with all 12 exons and encoding a 679-aa protein), and two truncating forms: LGP2v1 (lacking the exon 9 and encoding a 575-aa protein with incomplete DExDc domain) and LGP2v2 (lacking the exons 3 and 4 and encoding a 547-aa protein with incomplete HELIc domain) [102]. All three variants were up-regulated in ZFL cells during poly(I:C) stimulation and in immune-related organs of zebrafish during SVCV infection, showing differential expression patterns and higher expression level of *lgp2* than *lgp2v1* and *lgp2v2* [102].

Similarly to the situation observed in mammals, there are many reports indicating contrary biological activities of fish LGP2 as a positive or negative regulator of antiviral response. For instance, it was demonstrated for EPC cells that overexpression of zebrafish LGP2, but not overexpression of LGP2v1 and LGP2v2, significantly inhibited SVCV replication and expression of viral genes through induction of type I IFN and ISGs expression [102]. It was therefore concluded that LGP2 alone as well as under stimulation with low concentrations of poly(I:C) and at the early stages of SVCV infections functions as a positive regulator of IFN type I response, however, with the increasing concentrations of poly(I:C) or SVCV titer and consequent to the robust type I IFN response, it switches to negative function. LGP2v1 and LGP2v2, however, demonstrate only the inhibitory functions [102]. Ohtani and co-workers [57] demonstrated that overexpression of Japanese flounder LGP2 in HINAE cells has protective effect upon VHSV and HIRRV infection, while transfection of this cells with LGP2ΔRD failed to protect them from VHSV infection and provided only slight protection against HIRRV infection [57]. These studies also demonstrated that overexpression of full-length LGP2 in HINAE cells resulted in significant induction of type I IFN and ISGs (*mx*, *isg15*, and *isg*56) as compared to the cells transfected with LGP2ΔRD and empty plasmid during VHSV infection at 24 hpi [57]. In rainbow trout overexpression of *lgp2a*, but not *lgp2b*, in RTG-2 cells confers up-regulation of the expression of *mx* and enhances protection against VHSV [78]. Interestingly, an inhibitory effect of LGP2b on LGP2a was demonstrated [78]. It has been also shown in rainbow trout that both LGP2a and LGP2b, and MDA5 can bind poly(I:C) [78]. Chen and co-workers [118] described the contrary effect of grass carp *lgp2* overexpression during GCRV infection and viral or bacterial PAMPs stimulation on endogenous *rig-I* and *mda5* expression. Moreover, *lgp2* overexpression confers its inhibitory effect on GCRV replication and therefore, positive role in anti-GCRV immune response [118]. The positive role of LGP2 in antiviral immune response was also demonstrated in miiuy croaker (*Miichthys miiuy*) [98] and black carp (*Mylopharyngodon piceus*) [79,95]. It is also suggested that LGP2 acts as a positive regulator in MDA5 mediated signaling during viral infections [79,118].

There are also various reports suggesting that fish LGP2 acts as a negative regulator in the activation of the RIG-I/MDA5 signaling pathway. Yu and co-workers [99] demonstrated that overexpression of orange-spotted grouper LGP2 in GS cells induced an enhanced CPE and increased expression of viral genes during RGNNV and SGIV infection as compared to the control cells. Overexpression of fish LGP2 caused a marked decreased expression of transcription factors (*irf3*, *irf7,* and *nf-κB*), IFNs and ISGs in unstimulated cells [99,119], as well as in cells stimulated with poly(I:C) [87], and during early phase of GCRV infection [119]. Furthermore, Rao and co-workers [119] demonstrated that knock-down of *lgp2* increased phosphorylation levels of IRF3 and IRF7 and increased expression of type I IFNs in unstimulated cells but not in cells infected with GCRV. Interestingly LGP2 was shown to inhibit MAVS (IPS-1) activation by RIG-I and MDA5 via direct protein-protein interactions with both RLRs [119]. Moreover, in Nile tilapia (*Oreochromis niloticus*), LGP2 decreased MAVS-dependent NF-κB activation which suggests its negative regulatory effect on the MAVS gene [117].

## 5. Non-RLR DExD/H-box RNA Helicases

Besides RLR group, several non-RLR DExD/H-box RNA helicases have been described in mammals as proteins involved in sensing of viral genetic material and activation of downstream signaling pathways leading to type I interferon (IFN) production. They include: DDX1 [120], DDX3 [121,122], DHX9 [123], DHX15 [124], DDX17 [125], DDX21 [120], DDX23 [126], DHX29 [127], DHX36 [120], DDX41 [128] and DDX60 [129]. Interestingly, some of them, like DHX33, can also recognize bacterial RNA [130].

In mammals, various non-RLR DExD/H-box RNA helicases can recognize both pathogenic DNA and RNA and signal via distinct adaptor molecules (Figure 1A,B). For example, DDX1 was found to form a complex with two other DExD/H-box RNA helicases, DDX21 and DHX36, and in this complex, DDX1 directly binds to both short and long forms of poly(I:C) while DDX21 and DHX36 are responsible for signal transduction via TRIF adaptor molecule [120]. DHX9 was described as a cytoplasmic sensor for both dsRNA in murine myeloid dendritic cells [123] and CpG DNA in human plasmacytoid dendritic cells [131]. Depending on the nature of the ligand and the cell type, DHX9 induces signaling through MAVS adaptor molecule (upon dsRNA binding) or in a MyD88-dependent manner (upon DNA binding) [123,131]. Another RNA helicase, DDX3, was shown to interact with IKKε [121] or TBK1 [122] kinases and act as a signaling intermediate downstream of TBK1/IKKε in the IFN-β induction pathway. Moreover, activated by TBK1, DDX3 binds directly to the IFN-β promoter enhancer region, and functions as a transcriptional regulator [122]. Finally it was suggested that at the early stages of infection, DDX3 can also sensitize the RLR system for dsRNA ligands [132]. In mammals, DDX41 recognizes intracellular DNA derived from bacteria and induces the production of type I IFNs in myeloid dendritic cells (mDCs) in a STING-dependent manner [128,133].

Phylogenetic analysis of fish non-RLR DExD/H-box RNA helicases resulted in two main clusters: DEAD-box and DEAH-box [100,103,104,105]. Moreover, this analysis confirmed that motifs constituting these helicases were well conserved among species, however motif V was missing in DHX9 of common carp and in DHX32A and DHX32B of channel catfish [103,134]. Constitutive expression of all studied non-RLR DExD/H-box RNA helicases in different organs among studied fish species was demonstrated [100,103,104,105,107,135]. The intracellular localization of several non-RLR DExD/H-box RNA helicases was also studied. In trout RTG-2 cells, DDX3 was localized to the cytoplasm, while DHX9 was localized mainly in the nucleus [100]. DDX41 was localized in the nucleus of GS cells [107].

Compared to mammals, the role of non-RLR DExD/H-box RNA helicases in antiviral response of fish is poorly described. The expression of fish non-RLR DExD/H-box RNA helicases in response to DNA and RNA viruses, poly(I:C) stimulation as well as to bacterial infection and CpG was studied in different fish species [100,103,104,105,106,107]. The results of these studies are summarized in Table 1. Moreover, in rainbow trout, DDX3 and DHX9 were observed to bind dsRNA [100].

In fish, there are few functional studies of non-RLR DExD/H box RNA helicases. *Ddx3* overexpression in GS cells resulted in enhanced type I IFN-related antiviral response and inhibition of replication of RGNNV but not SGIV [104]. Overexpression of Japanese flounder *ddx41* induced activation of antiviral status of the HINAE cells upon C-di-GMP stimulation [106]. The overexpression of Nile tilapia DDX41 induced a strong activation of both zebrafish IFN1 and IFN3 promoters in EPC cells treated with poly(dA:dT) [136]. Furthermore, overexpression of *ddx41* in GS cells inhibited SGIV and RGNNV replication as well as increased the gene expression of antiviral and pro-inflammatory cytokines [107]. These results suggest that DDX41 is involved in the type I IFN-mediated antiviral and inflammatory response in teleosts.

## 6. MAVS

So far many MAVS orthologs have been detected in teleost fish [89,91,110,117,118,137,138,139,140,141,142,143,144,145,146]. It was observed moreover, that fish MAVS contains similar domains as the mammalian one [43] and is associated with both RIG-I and MDA5 signaling pathways, and can induce type I IFN response through activation of TRAF3 and TBK1 [43]. For example, grass carp MAVS induced activation of type I IFN through IRF7 but not IRF3 [141] while zebrafish MAVS splicing variant (MAVS_tv2) suppressed activation of type I IFN by targeting IRF7 [142]. Intracellular localization of MAVS in mitochondria was demonstrated in various fish species [110,137,145,147,148]. Interestingly Xiang et al. 2011 [139] reveal that MAVS of *Tetraodon nigroviridis* is located near by the plasma membrane but not merged with mitochondria in fathead minnow (FHM) epithelial cell.

Up-regulation of the expression of *mavs* after viral infection or poly(I:C) stimulation in cell lines (in vitro studies) or in various organs of different fish species (in vivo studies) have been widely demonstrated in many reports [64,91,140,144,147,149,150,151,152,153,154]. The functional studies demonstrated that inhibition of MAVS impairs the antiviral response in fish. For example, cytosolic poly (I:C)-induced or RIG-I-induced type I IFN response was attenuated by functional blockade of crucian carp MAVS [155]. Deletion of CARD or its transmembrane domains (TM) in Atlantic salmon abolished activation of the IFNa1 promoter and the NF-κβ driven promoter [137]. Similarly, deletion of the TM in MAVS of *Tetraodon nigroviridis* resulted in inhibition of ISRE and NF-κβ activation while deletion of the CARD domain negatively affected MAVS function on the ISRE but not affected activation of NF-κβ [139]. The protective effect of *mavs* overexpression against viral infections was manifested by reduced CPE and decreased viral titer or viral gene expression in studied cell lines of different fish species [110,138,140,145,147,149,152]. Moreover overexpression of *mavs* induced up-regulation of the expression of several downstream signaling molecules (e.g., *irf3* and *irf7*), type I IFNs and antiviral proteins [117,138,139,140,145,147,153,155] Interestingly, co-transfection of nile tilapia MAVS with MDA5 resulted in the slightly increased activation of MAVS-dependent NF-κB, whereas co-transfection of nile tilapia MAVS with LGP2 resulted in significantly decreased MAVS-dependent NF-κB activation [117].

## 7. cGAS

Cyclic guanosine monophosphare-adenosine monophosphate (GMP-AMP) synthase (cGAS) is one of the cytosolic PRRs that binds to double-stranded DNA (dsDNA) in the cytoplasm and catalyzes the synthesis of second messenger molecule 2′3′-cGAMP. cGAS belongs to the family of nucleotidyltransferase (NTase) enzymes [156].

In mammals, cGAS is composed of N-terminal and C-terminal male abnormal 21 (mab21) domains. The mab21 domain has a region that binds Zn^2+^. The coordinated Zn^2+^ (Zinc-Ribbon) binds across the dsDNA to form a cGAS-DNA complex, which excites the cGAS protein [156,157]. The Zn^2+^ binding region attaches to other NTase family proteins. Deletion of zinc-ribbon domain shows that it is importance in dsDNA sensing by cGAS [158]. Furthermore, the N-terminal domain of mammalian cGAS plays an important role in mounting the STING/IRF3-mediated cytosolic DNA signaling [159]. cGAS is conserved in various species spanning from fish to mammals. In teleosts, the N-terminal region of the cGAS protein is markedly conserved, while the amino acids involved in the DNA-binding surface and cGAMP synthesis in the mab21 domain are also highly conserved [105,160].

Upon binding to foreign DNA of viruses, bacteria, and parasites that invade into cells and upon detection in the cytoplasm of mislocalized endogenous DNA, cGAS catalyzes the synthesis of a second messenger molecule, cGAMP (from ATP and GTP) (Figure 1B). cGAMP binds to the protein stimulator of interferon gene (STING), which is localized in the endoplasmic reticulum (ER) membrane, and shifts translocation of STING from the ER to the Golgi apparatus. Next, STING promotes the activation of TANK binding kinase 1 (TBK1). Activated TBK1 causes phosphorylation and dimerization of the IRF3 or IRF7. These transcription factors translocate into the nucleus and induce production of type I IFNs. At the same time, STING promotes nuclear translocation of NF-κB, what induces the production of pro-inflammatory cytokines [161,162,163]. Furthermore, upon nonspecific binding of cytosolic B-form DNA, cGAS synthesizes the 2′3′-cGAMP and triggers STING-dependent signaling [159]. However, human cGAS favors DNA-length discrimination and sufficiently detects a longer DNA [164]. By transfecting THP-1 cells with dsDNA of 88 to 4003 bp at a DNA, a ligand length-dependent type I IFN response was confirmed [165].

The function of cGAS against pathogens has been extensively studied in mammals, but little is known about their functions in fish. In mammals, injection of genomic DNA of pathogens including viruses, bacteria, and parasites into monocyte-derived cells excites immune signals downstream of cGAS. The function of cGAS against pathogens including virus, bacteria and parasite in mammals and fish are summarized in Table 2.

Although, induction of type I IFN by cGAS sensing mechanism is more powerful than its induction by other DNA sensors, pathogens may inhibit cGAS activity as a part of pathogen evasion mechanisms. For example, UL41 protein of Herpes simplex virus 1 (HSV-1) may partially degrade cGAS mRNA, while VP22 directly acts on the cGAS protein to degrade it [166,167,168]. The naked relaxed-circular DNA of chronic hepatitis B virus (HBV) is sensed in a cGAS-dependent manner in human hepatocytes, however host cell recognition of viral nucleic acids is abolished during HBV infection, which suppresses cGAS expression and function in the hepatocytes [169]. In addition, cGAS senses cDNAs of retroviruses (i.e., RNA viruses), such as human immunodeficiency virus (HIV), Murine leukemia virus (MLV) and Simian immunodeficiency virus (SIV), which are synthesized by reverse transcription in the cytoplasm. Moreover, Dengue virus (DENV) cleaves STING on the ER membrane with a protease, which also blocks self-DNA detection. Then, some of the viral proteins reach the mitochondrial membrane, causing mitochondrial stress and subsequent mtDNA leakage. Upon recognition of mtDNA leaked into the cytoplasm, cGAS synthesizes the second messenger molecule cGAMP, and induces IFN production by STING activation in DENV-infected cells and signals to neighboring cells via gap junctions [170]. This functional mechanism of mammalian cGAS was also confirmed by infection with intracellular bacteria [171,172,173,174] and parasites [175,176,177] (Table 2). Sensing *Mycobacterium tuberculosis* (*Mtb*) DNA via the cGAS-STING pathway induces type I IFN and autophagy [172,173], however, these inductions do not contribute to host protection against *Mtb* infection in mammalian lung cells [171]. Human cGAS also senses the genomic DNAs of malaria inducer, *Plasmodium falciparum* and *Leishmania donovani* and induces type I IFN production [176,177].

On the contrary, the function of cGAS against pathogens in fish is poorly understood. Infection of HSV-1 in cGAS knock-down zebrafish had no effect on induction of *ifnφ1*, *isg15* and *viperin*. Rather, double-knockdown of two zebrafish DNA sensors, DDX41 and DHX9, almost abolished the induction of above-mentioned antiviral genes. Therefore, cGAS was hardly involved in the biological defense of zebrafish, and it was considered that cGAS evolved in the animal kingdom after fish [178]. Currently, specific activities of cGAS variants were determined using in vitro assay and RP-HPLC measurements of cGAMP production. Although, the zebrafish cGAS homologue has low amino acid identity to human cGAS (less than 35%), their similar activities were determined [179]. Liu and co-workers [160] reported that overexpression of zebrafish cGASa/b in HEK293T cells and zebrafish embryos significantly activated NF-κB and type I IFN signaling pathways in a STING-dependent manner, and that cGASa, but not cGASb, was involved in immunoglobulin Z-mediated mucosal immunity in gill-associated lymphoid tissue, suggesting differential functions between the two DrcGASs [160]. Interestingly, it was revealed that the ortholog of cGAS in grass carp, cGAS-like (cGASL) by interacting with STING down-regulates transcription of type I IFN gene and functions as a negative regulator of IFN response. It suggests interaction between cGAS and MITA-TBK1 complex, which may partly hinder the phosphorylation process mediated by TBK1 [180]. Moreover, it has also been reported that infection of Japanese medaka with the intracellular bacteria, *E. tarda* promotes increased expression of *cgas* gene in the intestinal tract [105]. In summary, fish cGAS, like mammals, induces STING-mediated production of type I IFN through NF-κB activation. On the other hand, it may be involved in humoral immune response mediated by immunoglobulin Z, which has not been confirmed in mammals. It is still controversial whether fish cGAS is effective to avoid infection with pathogens.

## 8. LSm14A

Sm-like protein homolog A (LSm14A; also known as RAP55) belongs to the RNA-binding proteins. It is a highly conserved protein and a component of processing body (P-body) involved in mRNA metabolism. LSm14 was first discovered in the oocytes of *Pleurodeles waltl* and *Xenopus laevis* as RAP55 (mRNA-associated protein of 55kDa) [188,189]. LSm14A is composed of a N-terminal LSm domain that is necessary for RNA binding, localization to processing (P)-bodies and translational control while in a C-terminal domain there are a DFDF box, an FFD-TFG box and RCG repeats responsible for targeting the proteins to P-bodies in mammals [189,190]. Several studies of the function of LSm14A in higher vertebrates indicated that it is a sensor for viral nucleic acids and performs a pivotal role in activation of IFN-β signaling pathway [191,192,193,194,195,196]. However, the function of LSm14A in teleosts is still very poorly understood. Recently, LSm14A has been identified in Japanese medaka [105]. The Japanese medaka Cab LSm14A cDNA is a 1311 bp long ORF encoding a predicted protein of 436 amino acids. Both the LSm and FDF domains in LSm14A are highly conserved, especially the sequence identities of medaka LSm domain were 96–99% to that of 14 other vertebrates [105]. Moreover, it was found that *lsm14a* gene was induced in the intestine, kidney, and spleen of formalin-killed *E. tarda*-treated medaka, what suggests that the transcription of this CDS gene was induced by the stimulation with dsDNA of *E. tarda*. As there is a single report so far, the identification and function of this molecule of other fish species require to be studied for further understanding.

## 9. PKR and PKZ

The double stranded RNA (dsRNA)-dependent protein kinase R (PKR) (alternatively called eukaryotic translation initiation factor 2-alpha kinase 2; E2AK2 or eIF2AK2) is one of the most studied interferon-induced proteins presented in all vertebrates [197,198,199,200]. PKR contains two N-terminal dsRNA binding domains and a catalytic kinase domain in the C terminal region. Its activation is triggered by binding to viral derived dsRNA, which resulted in homodimer formation and autophosphorylation. Active PKR contributes to the anti-viral innate immune response, mainly (but not exclusively) by phosphorylation of the eukaryotic initiation factor 2 alpha (eIF-2α) and consequent shut-down of protein synthesis [201]. Interestingly, a paralogue of PKR, called PKR-like protein kinase containing Z-DNA binding domain (PKZ) [202,203], which has a closer evolutionary relationship with PKR [204], has been identified in teleost fish species [203]. The C-terminal regions of PKR and PKZ are closely related (both encode an active protein kinase domain). In contrast, the N-terminal region of PKZ contains two Zalpha domains instead of the dsRNA binding domains found in PKR. Zalpha domains of PKZ bind the left-handed dsDNA (Z DNA) [205]. Binding of PKZ to Z-DNA induces its activation through homodimerization and autophosphorylation. Once activated PKZ, like PKR, is able to phosphorylate eIF-2α thereby blocking protein synthesis [203,206,207]. In addition to PKZ, Zalpha domains have only been identified in two additional cellular proteins which play a role in regulation of interferon responses: the RNA-editing enzyme ADAR1 (described in vertebrates) [208] and the Z-DNA binding protein 1 (ZBP1), also known as DNA-dependent activator of IFN-regulatory factors (DAI) or DLM-1 [209]. On account of the fact that DAI (ZBP1/DLM-1) has not been found in the fish genome, it can be hypothetized that PKZ is a kind of compensator for the lack of DAI and function as a cytosolic DNA sensor triggering innate antiviral immune response [210]. The antiviral functions of PKR and PKZ are completely different compared to the effect of the activation of RLRs that induce expression of the gene encoded the host antiviral factors.

In the fish of order cypriniformes, such as goldfish (*Carassius auratus*), grass carp (*Ctenopharyngodon idella*) and zebrafish (*Danio rerio*), *pkr* and *pkz* genes are tandemly arranged in head-to-tail (parallel) orientation on the same chromosome, with respect to 5′ to 3′ alignment of the genes [199,207,210]. In contrast, in the fish of order tetraodontiformes, such as spotted green pufferfish (*Tetraodon nigroviridis*) and Japanese pufferfish (*Takifugu rubripes*), two or three *pkr* genes (*pkr* 1, *pkr* 2, and *pkr* 3) are tandemly arranged on the same chromosome [199,203], suggesting that one of the *pkr* genes could be rearranged to *pkz* or an ancestral gene could be differently duplicated by some evolutional events between cypriniformes to tetraodontiformes. Fish *pkr* genes have been already cloned from many fish species; goldfish [207], grass carp [211], Japanese flounder (*Paralichthys olivaceus*) [200], Japanese pufferfish [212], rock bream (*Oplegnathus fasciatus*) [213], spotted green pufferfish [199], and zebrafish [214], while *pkz* genes have been identified in Atlantic salmon (*Salmo salar*) [206], Chinese rare minnow (*Gobiocypris rarus*) [215], goldfish [202,207], grass carp [216], and zebrafish [203] (Table 3). Fish *pkr* and *pkz* genes are constitutively expressed at a very low level in cultured cells and tissues and their expression is up-regulated in response to stimulation with interferon, poly(I:C), poly(dA-dT), poly(dG-dC), genomic DNA, and during infections with several viruses but also bacterial *Aeromonas hydrophila* [199,202,203,206,207,215,216]. It was moreover found that overexpression of *pkr* or *pkz* in transfected cells significantly inhibited protein synthesis [203]. Although the importance of PKR in antiviral innate immune response has been demonstrated by an extensive studies using various virus-host models, the anti-viral role of PKZ has been investigated to the small extend. For example [207] demonstrated that PKR and PKZ can cooperate roles in antiviral innate immune response against grass carp reovirus. The antiviral functions of PKR and PKZ in fish are summarized in Table 3.

## 10. Conclusions

The recognition of various kinds of intracellular nucleic acids of pathogens and initiation of innate immune responses to them by host cells has yet to be fully understood. Over the past decade or so, several investigations have focused on clarifying the mechanisms underlying these innate immune responses triggered by sensors that recognize pathogen-derived components in cytosol. The findings of these studies have suggested that in higher vertebrates immune responses to viral or bacterial nucleic acids are mediated by cytosolic sensors and their adaptor molecules while our understanding of this systems/molecules in other animals is less advanced. Teleost fish form a perfect model to identify and extend our knowledge of evolutionary conservation of important ligands and receptors involved in this process. They are the first species to utilize both an innate and adaptive response with specific antibodies to combat infections. Moreover, they are found in essentially every aquatic habitat and have been successful in adapting to different environments. More than half of all extant vertebrate species belong to this group (there are an estimated 35,000 species of bony fish) and their ability to recognize and eradicate pathogens must have contributed to this success.

It is already known that fish cells, similarly to their mammalian counterparts, respond to a variety of pathogens through their PRRs, which recognize different types of nucleic acids from pathogens. As in mammals also in fish, viral RNAs are recognized by DExD/H-box RNA helicases that include a group of well-characterized RLRs (RIG-I/DDX58, MDA5 and LGP2) and non-RLRs (DDX1, DDX3, DHX9, DDX21, DHX36, and DDX41). Schematic representation of the domain topology and conserved motifs of fish and mammalian RLRs and non-RLRs DExD/H-box RNA helicases revealed that the structure of these receptors is very conserved. The same domains and motifs are present in these receptors in fish and mammals [22,46,100]. Although, all the three RLRs (RIG-I, MDA5 and LGP2) are mostly conserved in fish, RIG-I does not exist in some fish species of Acanthopterygii superorder therefore it can be hypothesized that fish *rig-I* gene could has been lost after the divergence occurring before the Acanthopterygian fish [22]. In turn, among non-RLRs DExD/H-box RNA helicases, DDX1, DDX3, DHX9, DDX21, DHX36 and DDX41 have been described in several fish species and their role in antiviral and antibacterial immune response was confirmed. Also another RNA-binding cytosolic PRR, LSm14A/RAP55, described to act as a sensor for viral nucleic acids in higher vertebrates, it has recently been identified in fish with a potential ability to sensing dsDNA of an intracellular bacterium. In contrast function of teleost cGAS in the recognition of viral, bacteria and parasite-derived DNAs has not yet been elucidated clearly. Intriguingly, next to exerting antiviral immune responses upon sensing dsRNA, PKR, fish possess a novel PKR-like protein kinase containing Z-DNA binding domain, named as PKZ which is involved in sensing of Z-DNA.

Although, these cytosolic PRRs recognizing pathogenic viral and bacterial nucleic acids have recently been identified from various fish species, their detailed origin and evolution, involvement of adaptor molecules, exact ligand binding specificities, downstream signaling pathways with regulation mechanism, and induction of type I IFN and inflammatory cytokine production are largely unknown and may vary compared to those of mammals. Therefore, further studies are required to reveal these aspects clearly. Regardless of existence of differences in recognition mechanisms for cytosolic viral and bacterial nucleic acids between fish and other vertebrates, there is much similarity, suggesting an evolutionarily conserved immune system that is indispensable for pathogen sensing and removal.

More detailed recognition of this molecules and pathways will extend our knowledge but will have also practical aspect to design and improve new strategies for fish health control. This is of great importance in view of the annual growth of the fish farming industry, where infectious diseases have a significant impact on productivity and profitability.

## Figures and Tables

**Table 1:** The upregulation of the expression of RLR and non-RLR DExD/H-box RNA helicases induced by viral/bacterial infection or PAMPs stimulation in fish.

Species	Cell Line/Tissue	Infection/Treatment	References
***rig-I***			
**Atlantic salmon** (*Salmo salar*)	In vitro: TO cell line	salmon alphavirus subtype 3 (SAV-3), infectious pancreatic necrosis virus (IPNV), infectious salmon anaemia virus (ISAV)	[59,60]
	Ex vivo: erythrocytes	piscine orthoreovirus (PRV)	[61]
	In vivo: head kidney	infectious pancreatic necrosis virus (IPNV)	[62]
**Channel catfish** (*Ictalurus punctatus*)	In vitro: CCO cells line	channel catfish virus (CCV)	[63]
	In vivo: liver	*Edwardsiella ictaluri*	[63]
**Common carp** (*Cyprinus carpio*)	In vivo: spleen, head kidney, intestine	spring viremia of carp virus (SVCV)	[64]
**Gibel carp** (*Carassius gibelio*)	In vivo: head kidney, spleen, liver	*Carassius auratus* herpesvirus (CaHV)	[65]
**Grass carp** (*Ctenopharyngodon idella*)	In vitro: CIK cell line	grass carp reovirus (GCRV), poly(I:C)	[66]
	In vitro: primary trunk kidney cells	grass carp reovirus (GCRV), poly(I:C), LPS, PGN	[67]
	In vivo: spleen, liver, trunk kidney, hepatopancreas	grass carp reovirus (GCRV)	[66,67,68]
**Japanese eel** (*Anguilla japonica*)	In vivo: liver, spleen, kidney	poly(I:C), LPS, *Aeromonas hydrophila*	[69]
	In vivo: liver, spleen, head kidney, skin, intestine, and gills	poly(I:C)	[70]
**Zebrafish** (*Danio rerio*)	In vitro: ZF4 cell line	nervous necrosis virus (NNV), snakehead fish vesiculovirus (SHVV), spring viremia of carp virus (SVCV), *Edwardsiella tarda*	[71,72,73]
	In vivo: larvae	spring viremia of carp virus (SVCV), poly(I:C)	[74,75]
	In vivo: visceral tissues	poly(I:C)	[76]
	In vivo: spleen, kidney	tilapia lake virus (TiLV)	[77]
***mda5***			
**Atlantic salmon** (*Salmo salar*)	In vitro: TO cell line	salmon alphavirus subtype 3 (SAV-3), infectious pancreatic necrosis virus (IPNV), infectious salmon anaemia virus (ISAV), salmonid alphavirus	[59,60,78]
	In vivo: head kidney	infectious pancreatic necrosis virus (IPNV)	[62]
**Black carp** (*Mylopharyngodon piceus)*	In vitro: MPF cell line	spring viremia of carp virus (SVCV), grass carp Reovirus (GCRV), poly(I:C)	[79]
	In vivo: heart, liver, spleen, kidney, intestine, skin, gill	grass carp reovirus (GCRV), spring viremia of carp virus SVCV	[79]
**Asian seabass** (*Lates calcarifer*)	In vitro: SISK cell line	poli (I:C), LPS, PGN	[80]
	In vivo: spleen, kidney, gills, heart, liver, intestine	poli (I:C)	
	In vivo: gills, heart, kidney, liver, intestine	*Vibrio alginolyticus*	
	In vivo: gills, heart, kidney, liver, intestine	*Staphylococcus aureus*	
**Barbel chub** (*Squaliobarbus curriculus*)	In vivo: liver	grass carp reovirus (GCRV)	[81]
**Channel catfish** (*Ictalurus punctatus*)	In vitro: CCO cell line	channel catfish virus (CCV)	[63]
	In vivo: liver	*Edwardsiella ictaluri.*	[63]
**Common carp** (*Cyprinus carpio*)	In vitro: peripheral blood leukocytes	poly(I:C)	[82]
	In vivo: liver, spleen, head kidney, foregut, hindgut, gills, skin	poly(I:C), *Aeromonas hydrophila*	[82]
**Grass carp** (*Ctenopharyngodon idella*)	In vitro: primarly trunk kidney cells	grass carp reovirus (GCRV), poly(I:C), LPS, PGN	[67]
	In vivo: trunk kidney, spleen, liver, hepatopancreas	grass carp reovirus (GCRV)	[67,68,83]
**Green chromide** (*Etroplus suratensis*)	In vivo: spleen, kidney, liver, intestine, gills, heart	poly(I:C)	[84]
**Japanese flounder (Olive flounder)** (*Paralichthys olivaceus*)	In vitro: peripheral blood leukocytes, kidney leukocytes	poly(I:C), LPS	[85]
	In vivo: kidney	viral hemorrhagic septicemia virus (VHSV)	[85]
	In vitro: HINEA cell line	poly(I:C)	[86]
**Mandrinfish** (*Siniperca chuatsi*)	In vivo: spleen, gills, head kidney	poly(I:C), LPS	[87]
**Orange-spotted groupers** (*Epinephelus coioides*)	In vivo: spleen	Singapore grouper iridovirus (SGIV), poly(I:C)	[88]
**Rainbow trout** (*Oncorhynchus mykiss*)	In vitro: RTG-2 cell line, RTS-11 cell lines	poly(I:C), recombinant trout IFN2 protein	[78]
	In vivo: head kidney	viral hemorrhagic septicemia virus (VHSV)	[78]
**Sea perch** (*Lateolabrax japonicas*)	In vitro: LJB cell line, LJF cell line	Poly(I:C), nervous necrosis virus (NNV)	[89]
	In vivo: Spleen, kidney, eye, thymus, brain, intestine, muscle, gill, liver, heart	nervous necrosis virus (NNV)	
**Large yellow croaker** (*Pseudosciaena crocea*)	In vivo: peripheral blood, liver, spleen and head kidney	poly(I:C)	[90,91]
**Zebrafish** (*Danio rerio*)	In vitro: ZF4 cell line	spring viremia of carp virus (SVCV), nervous necrosis virus (NNV), snakehead fish vesiculovirus (SHVV), *Edwardsiella tarda*	[71,72,92]
***lgp2***			
**Asian seabass** (*Lates calcarifer*)	In vitro: SISK cell line	poly(I:C), LPS	[93]
	In vivo: liver, spleen, kidney, gill, heart, intestine	poly(I:C), *Vibrio alginolyticus*, *Staphylococcus aureus*	[93]
**Atlantic salmon** (*Salmo salar*)	In vitro: TO cell line	infectious pancreatic necrosis virus (IPNV), infectious salmon anaemia virus (ISAV), salmonid alphavirus (SAV)	[60,78]
**Atlantic cod** *(Gadus morhua)*	In vivo: head kidney	infectious pancreatic necrosis virus (IPNV)	[94]
**Black carp** (*Mylopharyngodon piceus)*	In vitro: MPF cell line	spring viremia of carp virus (SVCV), grass carp reovirus (GCRV), poly(I:C)	[95]
	In vivo: liver, spleen, kidney, intestine, heart, muscle, skin	spring viremia of carp virus (SVCV), grass carp reovirus (GCRV)	[95]
**Common carp** (*Cyprinus carpio*)	In vivo: muscle, spleen, gill, brain, skin, heart, intestine, liver, head kidney	koi herpes virus (KHV)	[96]
**Channel catfish** (*Ictalurus punctatus*)	In vitro: CCO cell line	channel catfish virus (CCV)	[63]
	In vivo: liver	*Edwardsiella ictaluri*	[63]
**Fathead minnow** (*Pimephales promelas*)	In vitro: EPC cell line	koi herpes virus, (KHV), poly(I:C)	[96]
**Gibel carp** (*Carassius gibelio*)	In vivo: head kidney, spleen, liver	*Carassius auratus* herpesvirus (CaHV)	[65]
**Grass carp** (*Ctenopharyngodon idella*)	In vitro: primary trunk kidney cells	grass carp reovirus (GCRV), poly(I:C), LPS, PGN	[67]
	In vivo: trunk kidney, spleen, liver, hepatopancreas	grass carp reovirus (GCRV)	[58,67,68]
**Indian major carp** (*Labeo rohita*)	In vitro: LRG cell line	poly(I:C), iE-DAP, MDP	[97]
	In vivo: liver, spleen, kidney, blood, gill	poly (I:C), *Aeromonas hydrophila*, *Bacillus subtilis*	[97]
**Japanese flounder (Olive flounder)** (*Paralichthys olivaceus*)	In vitro: leukocytes isolated from kidney	poly(I:C), LPS	[57]
	In vivo: kidney	viral hemorrhagic septicemia virus (VHSV)	[57]
	In vitro: HINEA cell line	poly(I:C)	[86]
**Large yellow croaker** (*Pseudosciaena crocea*)	In vivo: peripheral blood, liver, spleen, head kidney	poly(I:C)	[91]
**Mandrinfish** (*Siniperca chuatsi*)	In vivo: spleen, gills, head kidney	poly(I:C), LPS	[87]
**Miiuy croaker** (*Miichthys miiuy*)	In vitro: macrophages In vivo: liver, spleen, kidney	poly(I:C)	[98]
**Orange-spotted groupers** (*Epinephelus coioides*)	In vivo: spleen	Singapore grouper iridovirus (SGIV), poly(I:C)	[99]
**Rainbow trout** (*Oncorhynchus mykiss*)	In vitro: RTG-2 cell line, RTS-11 cell lines	poly(I:C), recombinant trout IFN2 protein	[78,100]
	In vivo: head kidney	viral hemorrhagic septicemia virus (VHSV)	[78]
**Sea perch** (*Lateolabrax japonicas*)	In vitro: LJF cell line, LJH cell line, LJB cell line	poly(I:C), nervous necrosis virus NNV	[101]
	In vivo: liver, heart, intestines, gill, spleen, muscle, brain, kidney, thymus, eye	nervous necrosis virus (NNV)	[101]
**Zebrafish** (*Danio rerio*)	In vitro: ZF4 and ZFL cell lines	poly(I:C), nervous necrosis virus (NNV), snakehead fish vesiculovirus (SHVV)	[71,72,102]
	In vivo: gill, liver, spleen, head kidney, and body kidney	spring viremia of carp virus (SVCV)	[102]
***ddx1***			
**Common carp** (*Cyprinus carpio*)	In vivo: head kidney	spring viremia of carp virus (SVCV)	[103]
**Zebrafish** (*Danio rerio*)	In vivo: kidney, spleen	spring viremia of carp virus (SVCV), chum salmon reovirus (CSV)	[103]
***ddx3***			
**Orange-spotted grouper** (*Epinephelus coioides*)	In vitro: GS cell line	poly(I:C), red-spotted grouper nervous necrosis virus RGNNV)	[104]
**Rainbow trout** (*Oncorhynchus mykiss*)	In vitro: RTG-2 cell line	poly(I:C)	[100]
**Zebrafish** (*Danio rerio*)	In vitro: ZF4 cell line	chum salmon reovirus (CSV)	[103]
***dhx9***			
**Common carp** (*Cyprinus carpio*)	In vivo: head kidney	spring viremia of carp virus (SVCV)	[103]
**Japanese medeka** (*Oryzias latipes*)	In vivo: spleen, kidney, intestine	CpG mixture, FKC of *Edwardsiella tarda*	[105]
**Rainbow trout** (*Oncorhynchus mykiss*)	In vitro: RTG-2 cell line	poly(I:C)	[100]
**Zebrafish** (*Danio rerio*)	In vitro: ZF4 cell line	chum salmon reovirus (CSV)	[103]
	In vivo: spleen	chum salmon reovirus (CSV)	[103]
***ddx21***			
**Common carp** (*Cyprinus carpio*)	In vivo: head kidney	spring viremia of carp virus (SVCV)	[103]
**Zebrafish** (*Danio rerio*)	In vitro: ZF4 cell line	chum salmon reovirus (CSV)	[103]
***dhx36***			
**Japanese medeka** (*Oryzias latipes*)	In vivo: spleen, kidney, intestine	CpG-mixture, FKC of *Edwardsiella tarda*	[105]
**Zebrafish** (*Danio rerio*)	In vitro: ZF4 cell line	chum salmon reovirus (CSV)	[103]
	In vivo: kidney	spring viremia of carp virus (SVCV)	
***ddx41***			
**Japanese flounder (Olive flounder)** (*Paralichthys olivaceus*)	In vitro: adherent (monocyte-like) and non-adherent (lymphocyte- enriched) cells	ranavirus	[106]
	In vivo: spleen, kidney, liver, heart, gill	lymphocystis disease virus (LCDV)	[106]
**Orange-spotted grouper** (*Epinephelus coioides*)	In vitro: GS cell line	Singapore grouper iridovirus (SGIV), red-spotted grouper nervous necrosis virus (RGNNV)	[107]

poly (I:C), polyriboinosinic:polyribocytidylic acid; LPS, lipopolysaccharide; PGN, peptidoglycan; iE-DEP, γ-D-glutamyl-meso-diaminopimelic acid; MDP, muramyl dipeptide; CpG mixture, unmethylated CpG DNA motif; FKC of *Edwardsiella tarda*, formalin-killed cells of *Edwardsiella tarda*.

**Table 2:** Function of cGAS against pathogenic virus, bacteria and parasite in fish and mammals.

Pathogens	Species	Function/Mechanism	References
**DNA viruses**			
African swine fever virus (ASFV)	**Pig (Wild boar)** (*Sus scrofa domesticus*)	ASFV occurs in attenuated and virulent forms. When avian alveolar macrophages are infected with attenuated ASFV, cGAS senses viral DNA and induces IFNβ. On the other hand, the virulent strain of ASFV strongly inhibits IFNβ *via* the cGAS-STING pathway by suppressing the downstream cascade of cGAS.	[166]
Herpes simplex virus 1 (HSV-1)	**Mouse** (*Mus musculus*)	Knockdown of cGAS by shRNA in murine fibrosarcoma cell line L929 strongly inhibits IRF3 dimerization induced by HSV-1 infection.	[167]
	**Human** (*Homo sapiens*)	HSV-1 tegument protein UL41 prevents cGAS DNA sensing by degrading cGAS mRNA.	[181]
		HSV-1 segment protein VP22 binds to cGAS protein and directly acts on the cGAS protein to degrade it.	[168]
	**Zebrafish** (*Danio rerio*)	The knockdown of cGAS did not cause an obvious effect on the induction of IFN-φ1, ISG15, and viperin in zebrafish infected with HSV-1.	[178]
Vaccinia virus Ankara (MVA)	**Human,** **Mouse**	Infection of human and mouse dendritic cells (DCs) with heat- or UV-inactivated MVA has been shown to induce higher levels of IFNs through the cGAS-STING pathway other than wild type-MVA.	[182]
		MVA DNAs in the cytosol are sensed by cGAS, what leads to activation of STING and downstream transcription factors, IRF3 and IRF7, resulting in the activation of type I IFN gene expression.	[182]
Chronic hepatitis B virus (HBV)	**Human,** **Mouse**	HBV rcDNA (rcDNA; a precursor of ccc) is sensed by cGAS in the cytoplasm of hepatocytes, but may form a viral capsid that covers the DNA and escapes from cGAS sensing. HBV cccDNA was increased in cGAS knockout cells and decreased in cGAS overexpressing cells.	[169]
		Constitutive low expression of cGAS-STING in the liver may explain liver-specific HBV infection and a weak capacity of hepatocyte cells to clear HBV infection.	[183]
Ectromelia virus (ECTV)	**Mouse**	Cells originated from bone marrow are the main type I IFN producers, required for ISG expression.	[184]
		In addition to TLR9, type I IFN production stimulated by the cGAS-STING pathway is also important for survival of mice after ECTV infection.	[185]
Human cytomegalovirus (HCMV)	**Human**	cGAS recognizes HCMV DNA and induces type I IFN in human monocyte-derived plasmacytoid dendritic cells and macrophages.	[186]
**RNA viruses (ssRNA)**			
Human immunodeficiency virus (HIV)	**Human,** **Mouse**	cGAS senses reverse transcribed HIV DNA in the cytoplasm and induces cGAMP-STING-dependent IFNβ production. Knockout or knockdown of cGAS in mouse or human cell lines blocks cytokine induction by HIV, MLV and SIV.	[187]
Murine leukemia virus (MLV)			
Simian immunodeficiency virus (SIV)			
Dengue virus (DENV)	**Human**	DENV infects cells and localizes to endoplasmic reticulum (ER) and mitochondrial membrane. Mitochondria are disrupted by infection stress, and cGAS senses mtDNA leaked into the cytoplasm.	[170]
**Intracellular bacteria**			
*Mycobacterium tuberculosis*	**Human,** **mouse**	The cGAS-STING pathway appears to activate dendritic cells by sensing Mycobacterium tuberculosis (Mtb) DNA in the cytoplasm, but does not contribute to host protection in vivo (lung cells).	[171]
		cGAS induces production of type I IFN and promotes early regulation of intracellular replication by inducing autophagy.	[172]
		Sensing Mtb DNA via the cGAS-STING pathway induces type I IFN and autophagy.	[173]
*Lysteria monocytogenes*	**Human**	Cell infected with *L. monocytogenes* DNA is carried to the extracellular vesicles (EV) of infected cells and delivered to bystander cells to stimulate the cGAS-STING pathway.	[174]
*Edwardsiella tarda* (*E. piscicida*)	**Japanese medaka** (*Oryzias latipes*)	*E. tarda* infection in medaka in vivo upregulated *cgas* gene expression.	[105]
	**Zebrafish** (*Danio rerio*)	DrcGASa contributed to IgZ/IgZ2 induction in response to *E tarda* infect/ion by upregulating *ifnφ1* expression in zebrafish gill γδ T cells.	[160]
**Extracellular bacteria**			
*Streptococcus pneumoniae*	**Human,** **Mouse**	The type I IFN response induced by mouse *S. pneumoniae* is highly dependent on the cGAS-STING pathway.	[175]
**Parasites**			
Malaria, *Plasmodium falciparum*	**Human**	cGAS is the cytosolic sensor of *P. falciparum* DNA and is required for the induction of IFNβ by malaria hemozoin (Hz) as carrier of *P. falciparum* gDNA.	[176]
*Leishmania donovani*	**Human**	cGAS dependent targeting of *L. donovani* DNA induces IFN-β over-production that contributes to antimony resistance in *L. donovani* infection.	[177]

ASFV, African swine fever virus; cGAS, cyclic GMP-AMP synthase; IFN, interferon; STING, stimulator of interferon genes; shRNA, short hairpin RNA; HSV-1, herpes simplex virus 1; ISG15, interferon-stimulated gene 15; DCs, dendritic cells; rcDNA, relaxed circular DNA; cccDNA, covalently closed circular DNA; MVA, modified vaccinia virus Ankara; HBV, hepatitis B virus; TLR9, toll-like receptor 9; ECTV, ectromelia virus; HCMV, human cytomegalovirus; HIV, human immunodeficiency virus; MLV, murine leukemia virus; SIV, simian immunodeficiency virus; DENV, dengue virus; *Mtb*, *Mycobacterium tuberculosis*; EV, extracellular vesicles; IgZ, immunoglobulin Z.

**Table 3:** Features and functions of PKR and PKZ in fish.

Fish Species	Superorder/Order	Features/Function	References
		**PKR**	
**Goldfish** *(Carassius auratus)*	Ostariophysi/Cypriniformes	Tandem arrangement of PKR and PKZ genes Induction of PKR mRNA by IFN-stimulation Phosphorylation of eIF2α Inhibition of translation Interaction with poly(I:C)	[207]
**Grass carp** *(Ctenopharyngodon idella)*	Ostariophysi/Cypriniformes	Induction of PKR mRNA by GCHV injection Inhibition of translation	[211]
**Japanese flounder (Olive flounder)** *(Paralichthys olivaceus)*	Acanthopterygii/Pleuronectiformes	Induction of PKR mRNA by SMRV injection Inhibition of translation Phosphorylation of eIF2α in response to SMRV infection	[200]
**Japanese pufferfish (torafugu)** *(Takifugu rubripes)*	Acanthopterygii/Tetraodontiformes	Tandem duplication of two PKR genes; Induction of PKR1 mRNA by poly(I:C) stimulation Inhibition of translation and NF-κB activation by PKR1 and PKR2	[199,212]
**Rock bream (Barred knifejaw)** (*Oplegnathus fasciatus*)	Acanthopterygii/Perciformes	Induction of PKR mRNA by poly(I:C)	[213]
**Spotted green pufferfish** *(Tetraodon nigroviridis)*	Acanthopterygii/Tetraodontiformes	Tandem duplication of three PKR genes	[199]
**Zebrafish** *(Danio rerio)*	Ostariophysi/Cypriniformes	Tandem arrangement of PKR and PKZ genes Phosphorylation of eIF2α	[199,207,214]
		**PKZ**	
**Atlantic salmon** *(Salmo salar)*	Protacanthopterygii/Salmoniformes	Induction of PKZ mRNA by IFN-stimulation Phosphorylation of eIF2α by PKZ-stimulated with Z-DNA in vitro	[206]
**Chinese rare minnow** *(Gobiocypris rarus)*	Ostariophysi/Cypriniformes	Induction of PKZ mRNA by GCRV and *Aeromonas hydrophila* infection	[215]
**Goldfish** *(Carassius auratus)*	OstariophysiCypriniformes	Induction of PKZ mRNA by IFN-stimulation;	[202,207]
**Grass carp** *(Ctenopharyngodon idella)*	Ostariophysi/Cypriniformes	Phosphorylation of eIF2α by PKZ in vitro Inhibition of translation	[216]
**Zebrafish** *(Danio rerio)*	Ostariophysi/Cypriniformes	Specific binding of Zα domains to Z-DNA Inhibition of translation Phosphorylation of eIF2α by both PKR and PKZ	[199,203]

eIF2α, eukaryotic initiation factor 2 alpha; GCHV, grass carp hemorrhage virus; SMRV, *Scophthalmus maximus* Rhabdovirus; GCRV, grass carp reovirus.
